# 

**Published:** 1786

**Authors:** 


### CATALOGUE OF BOOKS.

1. OBSERVATIONS on the Diſeaſes incident to Seamen. By *Gilbert Blane*, M. D. F. R. S. Phyſician Extraordinary to the Prince of Wales, Phyſician to St. Thomas's Hoſpital, and Phyſician to the Fleet in the late War. 8vo. *Murray*, London, 1786.

2. Introduction to the Practice of Midwifery, Part the Firſt. By *Thomas Denman*, M. D. Phyſician

Phyfician Man-midwife to the Middlefex Hofpital, and Teacher of Midwifery in London. 8vo. *Johnfon*, London, 1782 *.

3. An Effay on Uterine Hæmorrhages depending on Pregnancy and Parturition. By *Thomas Denman*, M. D. Licentiate in Midwifery of the College of Phyficians, and Teacher of Midwifery in London. 8vo. *Johnfon*, London, 1785.

4. An Effay on preternatural Labours. By *Thomas Denman*, M. D. &c. 8vo. *Johnfon*, London, 1786.

5. A Treatife on the Afthma. By *Thomas Withers*, M. D. Phyfician to the York County Hofpital. 8vo. *Robinfon*, London, 1786.

6. Obfervations on the Nature, Kinds, Caufes, and Prevention of Infanity, Lunacy, or Madnefs. By *Thomas Arnold*, M. D. Vol. II. Containing Obfervations on the Caufes and Prevention of Madnefs. 8vo. Leicefter, 1786.

7. Floræ Cantabrigienfis Supplementum. Auctore *Ricardo Relhan*, A. M. Collegii Regalis Capellano. 8vo. Cantab. 1786.

8. Obfervations on the Ufe of Crude Mercury, or Quickfilver, in Obftructions of the

* This valuable work, thongh printed in 1782, for the ufe of the author's pupils, was not offered for fale before the prefent year.

Bowels, arifing from Inflammation or other Caufes: With Remarks on the Ufe of Caftor Oil. By *R. S. Nevinfon*, Surgeon, Newark. 8vo. Newark, 1786.

9. Dr. Milman's Animadverfions on the Nature and on the Cure of the Dropfy. Tranflated from the Latin into Englifh, by *F. Swediaur*, M. D. 8vo. *Dodfley*, London, 1786.

10. The fingular Cafe of a Lady who had the Small-pox during Pregnancy, and who communicated the fame Difeafe to the Fœtus. By *W. Lynn*, Surgeon. 8vo. *Macrea*, London, 1786.

11. Tentamen Medicum Inaug. de Diarrhœa. Auctore *Joanne Addie*, Scoto. 8vo. Edin. 1786.

12. Differtatio Medica Inauguralis de Rachitide. Auctore *Gulielmo Barton*, Anglo. 8vo. Edin. 1786.

13. Differtatio Phyfica Inauguralis de Afcenfu Vaporum Spontaneo. Auctore *Samuele Black*, Hiberno. 8vo. Edin. 1786.

14. Differtatio Medica Inauguralis de Melæna. Auctore *Onuphrio Aram Bryam*, Britanno. 8vo. Edin. 1786.

15. Differtatio Medica Inauguralis de Variolis Infitivis. Auctore *Gulielmo Calder*, Britanno. 8vo. Edin. 1786.

16. Ten-

16. Tentamen Med. Inaug. de Chorea. Auctore *Joanne Ewart*, Scoto. 8vo. Edin. 1786.

17. Diſſertatio Medica Inauguralis de Hæmoptyſi. Auctore *Jacobo Forſter*, Hiberno. 8vo. Edin. 1786.

18. Diſſertatio Medica Inauguralis de Colica Saturnina. Auctore *Joanne Ffrye*, ex inſula Antiguæ. 8vo. Edin. 1786.

19. Tentamen Phyſiologico-Medicum Inaug. de Reſpiratione. Auctore *Henrico Gallaway*, Scoto. 8vo. Edin. 1786.

20. Diſſertatio Medica Inauguralis de Diarrhœa. Auctore *Jacobo Skelton Gilliam*, Virginienſi. 8vo. Edin. 1786.

21. Tentamen Inaug. de Electricitate Medica. Auctore *Gulielmo Haliday*, Hiberno. 8vo. Edin. 1786.

22. Tentamen Phyſiologico-Medicum Inaugurale de Menſibus et naturaliter et immodice fluentibus. Auctore *Biker Macdonald*, Hiberno. 8vo. Edin. 1786.

23. Diſputatio Medica Inauguralis de Suffocatione Stridula. Auctore *Georgio Monro*, Delavarienſi. 8vo. Edin. 1786.

24. Diſſertatio Medica Inauguralis de Scrophula. Auctore *Ricardo Pearſon*, Anglo. 8vo. Edin. 1786.

25. Diſſer-

25. Dissertatio Medica Inauguralis de Colica Pictonum. Auctore *Joanne Reid*, Scoto-Britanno. 8vo. Edin. 1786.

26. Tentamen Pathologicum Inaugurale de Causis Hydropum. Auctore *Gulielmo Robertson*, Scoto-Britanno. 8vo. Edin. 1786.

27. Tentamen Medicum Inaug. de Sanguinis detractionis usu et abusu. Auctore *Joanne Rogerson*, Scoto. 8vo. Edin. 1786.

28. Dissertatio Medica Inauguralis de Phthisi pulmonali a tuberculis oriunda. Auctore *Joanne Rutter*, Anglo. 8vo. Edin. 1786.

29. Tentamen Chemicum Inaugurale de Acido Atmospherico sive Aereo. Auctore *Gulielmo Scott*, Hiberno. 8vo. Edin. 1786.

30. Dissertatio Medica Inauguralis de Animo Demisso. Auctore *Caspar Wistar*, Pennsylvaniensi. 8vo. Edin. 1786.

31. Dissertatio Medica Inauguralis de Colica. Auctore *Gulielmo Younge*, Anglo. 8vo. Edin. 1786.

32. Observationes Botanicæ *. Auctore

* In this dissertation we meet with accurate descriptions of several new and rare plants cultivated in the Botanic garden, at Strasburgh. Among the engravings is an excellent figure of the *Salvia Leonuroides*, a Peruvian plant, the seeds of which were brought to Europe by M. Dombey.

*Benjam.*

*Benjam. Petro Gloxin*, Colmarienſi. 4to. Argentorati, 1785. c. tab. æneis 3.

33. De Morbis Cutaneis, ſpecimen 1. quo de Cruſta Lactea * adultorum agit, ac ſimul ad orationem ſolennem audiendam qua munus Profeſſoris Medicinæ extraordinarii d. 22. Junii, 1785, auſpicatus eſt, invitat *Joannes Henricus Fiſcher*, Medicinæ et Chirurg. Doctor et Profeſſor. 4to. Gottingæ, 1785.

34. Genera Morborum *Culleni*, juxta quartam ac noviſſimam Noſologiæ methodicæ editionem, Prælectionum uſui accomodata ab *I. H. Fiſcher*, M. D. ejuſgue in Acad. Gott. Prof. 8vo. Gottingæ, 1786.

35. Diſputatio Inauguralis Medica de Variolis Catarrhalibus. Auctore *Carolo Michaele Grummert*, Muſcovia Ruſſo. 4to. Gottingæ 1786.

36. Geſchichte der Luftgütepruefungslehre, *i. e.* Hiſtory of the means of aſcertaining the goodneſs of air, by *John Andrew Scherer*, M. D. 8vo. 2 vols. Vienna, 1784-5.

* The cutaneous diſeaſe to which different writers have given the name of Cruſta Lactea is a ſpecies of Tinea, and as ſuch is deſcribed by Sauvages in his *Noſologia Methodica*. In the work before us we have a curious and inſtructive account of a caſe of this kind in an adult, ſuccesfully treated by the author. Dr. Fiſcher means, on ſome future occaſion, to extend his inquiries to other diſeaſes of the ſkin.

37. Verfuch einiger praktifchen Anmerkungen uber die Eingeweide, zur Erlæuterung verfchiedner verbogener Krankheiten und Zufælle; *i. e.* An Effay, containing practical Obfervations on the Vifcera of the human Body, tending to illuftrate feveral of their more abftrufe Difeafes and Symptoms. By *Jacob Frederick Ifenflamm*, M. D. 8vo. Erlangen, 1784.

38. Abhandlung von der Schwind-Lungenfucht und den Mitteln wider diefelbe; *i. e* A Treatife on Pulmonary Confumption, and its Remedies. By *J. M. Marx*, M. D. Phyfician to the Electoral Court of Hanover. 8vo. Hanover, 1784.

39. Von dem auzfetzenden Puls, einigen andern Pulfarten und Angelegenheiten des Herzens. Eine femiotifch-praktifche Erlæuterungfchrift vom geheimen Hofrath und Profeffor *Delius*; *i. e.* On the intermitting Pulfe, and fome other Kinds of Pulfe, and Affections of the Heart. A Semeiologo-practical Effay by the Privy Counfellor and Profeffor *Delius*. 8vo. Erlangen, 1784.

40. Traité* de la Cataracte, avec des Obfervations

* This work contains many judicious obfervations on the cataract, together with a candid account of the mode of operating

ſervations qui prouvent la Neceſſité d'inciſer la Cornée tranſparente et la Capſule du Cryſtallin, d'une maniere diverſe, ſelon les differentes Eſpeces de Cataractes. Par M. *de Wenzel*, Fils, Baron du S. Empire, Medecin de la Faculté de Nancy, et Docteur Regent de la Faculté de Medecine en l'Univerſité de Paris. 8vo. Paris, 1786.

41. Recherches ſur les Moyens de prevenir la Petite Verole naturelle; et Procédés d'une Société établie à Cheſter pour cet Objet, et pour rendre l'Inoculation générale. Traduit de l'Anglois de M. *Haygarth*. Par M. *De la Roche*, Medecin de Monſeigneur le Duc d'Orleans, &c. 8vo. Paris, 1786.

42. Sulla formazione del Molibdeno, lettera di *Guil. Candida*, al Sign. *Vinc. Petagna*. 8vo. Napoli, 1785.

43. Nuovo Metodo di Medicare alcune Malattie ſpettanti alla Chirurgia, diviſo in quattro Diſſertazioni, a cui precedono gli Elogi Storici di Carlo Guattani, e di Pietro Maria Giavina, con la deſcrizione di ſue ſingolari Oſſervazioni, Chirurgica l'una ed Anatomica l'altra; di *Gui-*

rating adopted by the author's father, and figures of the inſtruments he employs.

*ſeppe Flajani*, Dottore di Filoſofia e Medicina, Chirurgo della Santita di Noſtro Signore Papa Pio VI. Lettore, Litotomo, e Chirurgo primario nell' Apoſtolico arciſpedale di Santo Spirito in Saſſia; Autore e Direttore del Muſeo Anatomico di detto Arciſpedale. 4to. Roma, 1786.

44. Medicina Teorica e Pratica ſopra la Malattia Contagioſa del Vajuolo; opera del Dottor *Andrea Volpe*, Medico Napoletano. 4to. Napoli, 1786.

45. Sovra il Contagio della Tiſichezza: Diſſertazione offerta al celebre e dotto Prelato, medico-fiſico Monſign. *Natale Saliceti*, archiatro del ſommo Pontefice felicemente regnante Pio VI. del Dottore *Mariano Narducci*, Medico-fiſico Macerateſe, ſocio delle Academie fiſico-botanica e dei georgofili di Firenze, &c. 8vo. Perouſe, 1785.

46. Deliciæ floræ et faunæ Inſubricæ ſ. novæ aut minus cognitæ ſpecies plantarum et animalium, quas in Inſubria Auſtriaca tam ſpontaneas, quam exoticas vidit, deſcripſit et aeri incidi curavit *Joannes Antonius Scopoli*, P. 1. fol. maj. Pavia, 1786.

INDEX.

### I N D E X.

A.

ACADEMY, American, Memoirs of, — Page 287

Adair, Dr. James Makittrick, Medical Cautions, 326

Addie, J. de Diarrhœa, — — 436

Aitken, Dr. J. Principles of Midwifery, — 222

Allioni, Carol. Flora Pedemontana, — — 333

Amputation, Obſervations on, — — 225, 377

Aneuriſm, of the Popliteal Artery, Treatment of, — 391

————————, Femoral Artery, Caſe of, — 401

Arbuſtrum Americanum, — — 103

Archer, Nicol. de Dyſenteria, — — 329

Aretæus, on Diſeaſes, Engliſh Tranſlation of, by Dr. Moffat, 103

Arnold, Dr. Tho. on Inſanity, — — 435

Arſenic, by whom recommended in Intermittents, — 192

Aſa Fœtida, a Plant yielding it deſcribed, — 68

Azille, M. D', ſur les Maladies des Climats chauds, — 224

B.

Banks, Sir Joſ. Remarks on a Plant that yields Aſafœtida, 71

Bark, its Solubility ſuppoſed to be increaſed by Magneſia, 254, 419

——, Eſſential Salt of, what ſo called, — 262

Barrow, Joannes, de Catarrho, — — 329

Barton, Gul. de Rachitide, — — 436

Bergman, Sir T. on elective Attractions, — 222

Binney, Barn. Caſe of a Gun-ſhot Wound, — 294

Birch, John, Caſe of an Aneuriſm of the femoral Artery, 401

Biſmuth, Magiſtery of, recommended as an Antiſpaſmodic, 321

Black, S. de Aſcenſu Vaporum Spontaneo, — 436

Blane, Dr. Gilbert, on the Diſeaſes of Seamen, — 434

Blunt, Robert, of a painful Affection of the Face, — 115

Bolton, James, Filices Britannicæ, — — 191

Bonn, And. Tabulæ Oſſium Morboſ. — — 333

Boſquilion, M. Elemens de Medecine pratique de M. Cullen, 105

Bouſſelin, M. on Necroſis, — — 263

Brandiſh, Joſ. Caſe of a Gun-ſhot Wound, — 135

Bryam, O. A. de Melæna — — 436

Bueno, Pedro Gut. ſobre las Aquas Minerales, — 223

Burns and Scalds, new Method of curing, — 103

C.

Calder, Gul. de Variolis inſitivis, — 436

Caſcaron, Don F. X. de, ſobre la Gonorrea, — 106

Caſpari, P. A. de Scilla, — — 336

Cavallo, Tiberius, Mineralogical Tables, — 104

Cauſer, John, Caſe of a Fracture of the Scull, — 152

Cawley, Thomas, Account of the Dyſentery, — 337

Chamſeru, M. Caſe in which the Colour of the Skin was changed, 280

Chavaſſe, N. on the Medical and Surgical Uſes of cold Water, 123

———————— a remarkable Diſeaſe of the Heart, — 407

Cheſelden, Mr. was acquainted with the Necroſis, — 264

Chorea Sancti Viti, Caſe of, — — 187

Clark, J. F. de Apoplexia, — — 329

Page
Clarke, John, two Cafes of laborious Parturition, — 40
Cline, Henry, Diffection of an Aneurifm, — 405
Clubbe, J. on the Gonorrhœa, — — 102
Coleman, William, Cafe of Worms difcharged through a Wound, 251
Cook, R. remarkable Cafe of Dropfy, — 54
Cofte, P. L. U. de Nervis, — — 335
Covey, John, Obfervations relative to Inoculation, — 180
Croft, Richard, Method of reducing the Funis, — 38
Crotchet, its Ufe in Cafes of narrow Pelvis, — 40
Cullen, Dr. French Tranflation of his Firft Lines of Phyfic, 105
Cuprum Ammoniacum, its Efficacy in Chorea Sancti Viti, 187

D.

Dawfon, J. Facts relative to the Small-pox, — 66
Delius, Profeffor, Von dem auzfetzenden Puls, — 440
Demetigny, M. L. de Natura Animæ et Corporis, - 336
Denman, Dr. Thomas, on the Ufe of the Globe peffary, - 56
————————, Introduction to the Practice of Midwifery, 434
————————, on Uterine Hæmorrhages, — 435
————————, on preternatural Labours, — *ibid.*
Dropfy, remarkable Cafes of, — — 54, 189
————, Contents of the Medullary Cells in, — 157
Duncan, Dr. Andrew, Medical Commentaries, — 326
————, Joannes, de Catarrho Epidemico, — 329
Dyfentery, Obfervations relative to, — 337

E.

Eifenlohr, T. G. de Hydrope Cyftico, — 108

Electricity, its Efficacy in Retention of Urine, — 10

———————————— a difeafed Tefticle, — 247

———————————— a painful Affection of the Face, 115

————————, recommended in the Cataract, — 141

Ewart, J. de Chorea, — — 437

F.

Face, painful Affection of, cured by Electricity, — 115
Fæces, hardened, Cafe of, — — 26
Fairclough, Edv. de Pertuffi, — — 329
Fevers, Ufe of cold Bathing in, — — 109
Ferris, Dr. S. Differtation on Milk, — — 102
Fifcher, J. H. de Morbis Cutaneis, — 439
————————, Genera Morborum, — *ibid.*
Fifher, Jofhua, Cafe of Tumor in the Abdomen, — 287
Flajani, G. Nuovo Metodo di Medicare alcune Malattie, 441
Forfter, Jac. de Hæmoptyfi, — — 437
Fothergill, Dr. Ant. on the Cheltenham Water, — 100
Fowler, Dr. Thomas, on the Effects of Arfenic in Agues, 192
Friccius, M. his Praife of Arfenic in Intermittents, — 194
Ffrye, J. de Colica Saturnina, — 437
Funis, new Method of reducing it, — — 38

G.

Gallaway, H. de Refpiratione, — — 437

Galley, Tho. de Tuffi Convulfiva, — — 223

Gilliam, J. S. de Diarrhœa, — — 437

Gloxin, B. P. Obf. Botanicæ, — — 438

Page

Gout, Treatiſe on, — — — 103

Grillot, Bern. de Vomitu Cruento, — — 335

Grummert, C. M. de Variolis, — — 439

H.

Haire, Lancelot, Remarks on Amputation, — 377

Haliday, Gul. de Electricitate Medica, — 437

Hall, John, on the Contents of the Medullary Cells in Dropſy, 157

Ham, J. P. de Fonticulorum uſu, — — 108

Hamilton, Dr. R. on the Hydrophobia, —— 89

————————, Caſe of Worms diſcharged through the Navel, 372

Haygarth, Dr. French Tranſlation of his Work on the Small-pox, 441

Hayle, Gul. P. de Cantharidibus, —— 331

Heart, Malconformation of, Caſes of, —— 280, 284

Heat, Animal, Obſ. and Queries relative to, — 169

Hedwig, Joannes, de Gener. Plant. Cryptog. —— 107

———————, de Stirp. Cryptogamic. — 333

Hemming, J. de Somno, —— —— 223

Higgins, Dr. Bryan, Experiments and Obſ. by, — 327

Hoffmann, G. F. Enumeratio Lichenum, —— 224

Holman, Charles, Caſe of Phthiſis unexpectedly relieved, 120

Home, Everard, on the Popliteal Aneuriſm, —— 391

Hope, Dr. J. Deſcription of a Plant yielding Aſafœtida, 68

Horn Diſtemper, in Cattle, deſcribed, — — 305

Houlſton, Dr. Tho. Experiments with variolous Matter, 7

Houndsfield, Geo. on the Cure of diſeaſed Teſtes by Electricity, 247

Hunter, John, Caſe of Hydrophobia, — — 89

——————, Treatiſe on the Venereal Diſeaſe, —— 205

——————, his Operation for the Popliteal Aneuriſm deſcribed, 391

Hydrophobia, Obſervations relative to, —— 81, 89

J.

Jenner, J. C. Caſe of Excreſcence in the Urethra, - 160

——————, Account of a general Inoculation at Painſwick, 163

Inoculation, Obſervations relative to, - 7, 63, 66, 163, 180

Introductio in Oryctrograph. Arragoniæ, —— 106

Johnſton, Carol. de Leucorrhœa, — — 223

Johnſtone, Dr. J. Caſe of a Gun-ſhot Wound, — 135

——————, Account of a remarkable Effect of Opium, 136

Journal, London Medical, French Tranſlation of, - 98

Irving, Dr. Ralph, Edinburgh New Diſpenſatory, - 325

——————, de Emeticis, — — 329

——————, on the ſolvent Power of Magneſia, - 419

Iſenflamm, J. F. Uber die Eingeweide, — 440

K.

Kæmpfer, the Fidelity of his Deſcriptions vindicated, — 71

Keir, Dr. W. Caſe of a fatal Vomiting, —— 72

Kentiſh, Dr. R. on a new Species of Bark, —— 99

Kirkland, Dr. Thomas, on the Uſe and Abuſe of Mercury, 1

Kirwan, Richard, Elements of Mineralogy, —— 99

Kite, Charles, on the Uſe of Electricity in the Cataract, 141

Koſegarten, D. A. J. F. de Camphora, —— 224

Kramp, Chriſtianus, de vi Vitali Arteriarum, — 335

L. Page

Lamoureux, J. Nic. An dolori Colico Opium? — 334

Lauth, Thomas, de Aneuryſmatibus, — 104

Leigh, Dr. J. on the Properties of Opium, — 327

Lind, Dr. J. on the Efficacy of Flowers of Zinc in Epilepſy, 52

Linné, Carol. von, Amænitates Academicæ, — 224

Liver, Abſceſs of, after a Blow, — — 22

Lucas, James, his Obſervations on Amputation, — 225

——————————, controverted, 377

Lues Venerea, known in the South-Sea iſlands before their Diſcovery by Cook, — — — 328

Lynn, W. Caſe of Small-pox during Pregnancy, - 436

Lyons, Jacobus, de Cholera, — — 329

M.

Macay, Samuel, de Hepatis Inflammatione, — 329

Macdonald, Biker, de Menſibus, — 437

Magneſia, its Effects as a Solvent of Bark aſſerted, - 254

—————————— controverted, - 419

Magnetiſm, Animal, Letter on, — 327

Meguire, Laur. White, Caſe of a Lumbar Abſceſs, — 14

Mann, Anton. de Diabete, — — 330

Marchand, N. D. de Ræſorbtione læſa, — 335

Mariotti, Annibale, delle Parotidi, — 333

Marſhall, H. Arbuſtrum Americanum, — 104

Marx, J. M. Von der Schwind-Lungenſucht, — 440

Maſdevall, Joſeph, Relacion de las Epidemias, — 334

Maſuyer, M. his Tranſlation of the London Medical Journal, 98

——————, ſur la Cure de la Gonorrhée, — 105

Mercury, ſmall Doſes of, in Syphilis, recommended, — 1

Meurs, L. Van, de Syſtemate Vaſorum Abſorbentium, - 331

Mignani, Vin. de Effectibus Terræ Motus, — 224

Milk, Diſſertation on, — — 102

Milman, Dr. Animadverſions on Dropſy, — 436

Moffat, Dr. J. Tranſlation of Aretæus, — 103

Monro, Dr. Alex. on the Structure of Fiſhes, — 100

Monro, Geo. de Suffocatione Stridula, — 437

Moore, Dr. J. Medical Sketches, — — 223

Moorhouſe, Henricus, de Aſcite, — — 320

Mortality, Obſervations on ſome American Bills of, - 316

Morton, Dr. David, Caſe of a Woman who performed the Cæſarean Section on herſelf, — — 61

N.

Narducci, Mar. Sovra il Contagio della Tiſichezza, - 442

Navel, Cautions with reſpect to the Treatment of, in Infants, 376

Necroſis, Obſervations on, — — 263

Nevinſon, R. S. on the Uſe of Crude Mercury, - 435

O.

Odier, Dr. L. on the Magiſtery of Biſmuth, — 321

Oliphant, Iſaac, Caſe of an Abſceſs of the Liver, — 22

—————————— hardened Fæces, — 26

—————————— Incontinence of Urine, — 416

Opium, remarkable Effect of, — — 136

Page
Ortega, Don C. G. Curfo de Botanica, — — 107
——————— Elogio de Don Jos. Quer, — — 335

P.

Painfwick, Account of a general Inoculation at, — 163

Parke, Mr. his Practice in difeafed Joints commended, — 226

——————————— objected to, — 383

Pearfon, Dr. Geo. Directions for imitating the Buxton Water, 102

———, John, on Animal Heat, — — 169

———, Ric. de Scrophula, — — 437

Petagna, V. del Molibdeno, — — 441

Pew, Richard, Medical Sketches, — — — 101

Pharmacopeia Wirtenbergica, — — — 108

Picardet, Madame, French Tranflation of Mr. Scheele's effays, ibid.

Pichon, C. A. F. P. de Ictero, — — 335

Pinel, M. Inftit. de Med. pratique de M. Cullen, — 105

Pitchel, F. L. Anatom. and Chirurg. Anmerkungen, — 106

Prieftley, Dr. Jof. Exp. and Obf. on various Branches of Natural Philofophy, — — — 222

Puig, S. Sobre las tercianas, — — 334

Puttmann, F. I. de ufu aquæ frigidæ, — — 108

Pyne, Cornel, de Rachitide, — — 330

Q.

Quer, Jofeph, Elogio hiftorico de, — — 335

R.

Reid, J. de Colica Pictonum, — — 437

Relhan, R. Floræ Cantab. Supplem. — 435

Remington, Tho. de Quatuor Vitæ Gradibus, — 223

Rigby, Edward, on Animal Heat, — — 100

Robertfon, G. de Caufis Hydropum, — 438

Roche, M. de la, fur la Petite Verole, — 441

Rodgers, I. R. B. de Dyfenteria, — — 330

Rogerfon, J. de Sanguinis Detractione, — 438

Rollo, Dr. I. on the Effects of drinking Spirits, — 33

Roux, M. le (Chirurgien) Differtation fur la Rage, — 81

——————— (Medecin) fur les Maladies des Ouvriers de la Digue, 336

Rufh, Dr. on the Difeafes of the American Army, — 76

——————— Tetanus, — — — 424

Rutter, J. de Phthifi Pulmonali, — 438

S.

Samwell, David, Obf. relative to the Lues, — 329

Sandifort, Edv. Defcriptio Offium Hominis, — 104

Saw, proper for Amputation, defcribed, — 237

Scheele, C. W. Memoires de Chymie, — 108

Scherer, J. And. der Luftguetepruefungflehre, — 439

Scopoli, J. A. Flora et Fauna Incubrica, — 442

Scott, Gul. de Acido Aereo, — — 438

Scurvy, curious Fact relative to its Generation, — 342

Simes, Robertus, de Rheumatifmo acuto, — 330

Simmons, Dr. S. F. Tranflations of his Work on the Gonorrhœa, 105, 106, 331

Skeete, Dr. Tho. Exp. and Obf. on Peruvian Bark, 254, 326

Small-pox, Facts relative to, — 7, 63, 66, 163, 180

Smith, Jac. Edv. de Generatione, — 331

Page

Snowden, Dr. S. Cafe of Retention of Urine cured by Electricity, 10

Spence, Dr. D. Treatife of Midwifery, — 221

Spirits, immoderate Ufe of, their Effects, — 33

Stender, R H. de Antimonii ufu Medico, — 336

Swediaur, Dr. F. Tranflation of Dr. Milman's Work on the Dropfy, —— —— 436

T.

Teeth, tranfplanted, Difeafe occafioned by, — 205, 216
Tenon, M. des Obftacles aux Progrés de l'Anatomie, — 224
Tefticle, difeafed, cured by Electricity, — 247
Thigh Bone, Head of, fuppofed to have been regenerated, 135
Throckmorton, Carol. de Sanguinis motu et quantitate, 330
Thunberg, C. P. Flora Japonica, —— 107
Trotter, Tho. Obfervations on the Scurvy, — 222
Tufts, Hon. Cotton, on the Horn Diftemper in Cattle, 305
Turnbull, W. on the Origin of the Lues, — 328

V.

Vaughan, Gualterus, de Connubio Chemico, — 331
Vidart, Pedro, Arte de Partear, — — 334
Volpe, And. fopra la Malattia del Vajuolo, — 442

U.

Urethra, female, Excrefcence in, — — 160

Ufher, Joannes, de Alimentorum Concoctione, — 330

W.

Wallis' Dr. G. on Difeafes of the Eyes, — 101
Warren, John, Cafe of a Tumor containing Hair, - 298
Water, cold, Obfervations on its medical Ufes, 109, 123
Watfon, Dr. William, on the Effects of tranfplanted Teeth, 216
Webfter, Dr. Charles, Edinburgh New Difpenfatory, — 325
Wenzel, M. de, Traité de la Cataracte, — 440
White, Dr. R. prefent Practice of Surgery, — 327
Willan, Dr. Rob. Cafes by, —— 187, 189
Wilfon, J. de Dyfenteria, — — 223
Wiftar, C. de Animo Demiffo, — — 438
Wltczek, Ign. Cafus Peculiaris Hiftoria, —— 332
Worms difcharged through Wounds communicating with the Inteftines, Cafes of, — — 251, 372
Wright, Dr. W. Facts relative to the Small-pox, — 63
——————, on Cold Bathing in Fevers, — 109
Wrigglefworth, Edw. on fome American Bills of Mortality, 316
Wy, J. Van, Heelkungige Mengelftoffen, — 106

Y.

Younge, Gul. de Colica, — — 438

Yule, Joannes, de Halitu Cuticulari, — 331

Z.

Zandycke, Dr. F. A. Van, Op het genezen van de Gonorrhœa, 331
Zinc, Flowers of, their Efficacy in Epilepfy inftanced, - 52

